# LAMP assays for the simple and rapid detection of clinically important urinary pathogens including the detection of resistance to 3rd generation cephalosporins

**DOI:** 10.1186/s12879-021-06720-5

**Published:** 2021-10-06

**Authors:** Lalainasoa Odile Rivoarilala, Jeannoda Victor, Tania Crucitti, Jean Marc Collard

**Affiliations:** 1 Experimental Bacteriology Unit, Institut Pasteur de Madagascar, Antananarivo, Madagascar; 2 University of Antananarivo, Antananarivo, Madagascar; 3 Present Address: Experimental Bacteriology Laboratory, Center for Microbes, Development and Health (CMDH), Institut Pasteur of Shanghai/Chinese Academy of Sciences, Shanghai, People’s Republic of China

**Keywords:** Loop-mediated isothermal amplification (LAMP), Urinary tract infection, Rapid diagnosis, CTX-M, Resistance gene

## Abstract

**Background:**

Timely and accurate identification of uropathogens and determination of their antimicrobial susceptibility is paramount to the management of urinary tract infections (UTIs). The main objective of this study was to develop an assay using LAMP (Loop mediated isothermal amplification) technology for simple, rapid and sensitive detection of the most common bacteria responsible for UTIs, as well as for the detection of the most prevalent genes (encoding cefotaximases from CTX-M group 1) responsible for resistance to 3rd generation of cephalosporins.

**Method:**

We designed primers targeting *Proteus mirabilis*, while those targeting *Escherichia coli*, *Klebsiella pneumoniae* and *Enterococcus faecalis* and the CTX-M group 1 resistance gene were benchmarked from previous studies. The amplification reaction was carried out in a warm water bath for 60 min at 63 ± 0.5 °C. The amplicons were revealed by staining with Sybr Green I. Specificity and sensitivity were determined using reference DNA extracts spiked in sterile urine samples. The analytical performance of the assays was evaluated directly on pellets of urine samples from patients suspected of UTI and compared with culture.

**Results:**

We found a high specificity (100%) for LAMP assays targeting the selected bacteria (*P. mirabilis*, *E. coli*, *K. pneumoniae*, *E. faecalis*) and the CTX-M group 1 when using DNA extracts spiked in urine samples. The sensitivities of the assays were around 1.5 10^3^ Colony Forming Units (CFU) /mL corresponding to the cut-off value used to define bacteriuria or UTIs in patients with symptoms. Out of 161 urine samples tested, using culture as gold standard, we found a sensitivity of the LAMP techniques ranging from 96 to 100% and specificity from 95 to 100%.

**Conclusion:**

We showed that the LAMP assays were simple and fast. The tests showed high sensitivity and specificity using a simple procedure for DNA extraction. In addition, the assays could be performed without the need of an expensive device such as a thermal cycler. These LAMP assays could be useful as an alternative or a complementary tool to culture reducing the time to diagnosis and guiding for more effective treatment of UTIs but also as a powerful diagnostic tool in resource-limited countries where culture is not available in primary health care structures.

**Supplementary Information:**

The online version contains supplementary material available at 10.1186/s12879-021-06720-5.

## Background

Urinary tract infections (UTIs) are among the most prevalent bacterial infections. They are a frequent reason for medical consultation and antimicrobial prescription in general medicine, ranking second after respiratory infections [1, 2]. UTIs are mainly caused by Enterobacteriaceae, with a predominance of *Escherichia coli*, responsible for about 90% of the community acquired UTI and up to 65% of the UTI acquired in hospitals [3, 4]. During the period of 2002 to 2008, the bacterial UTIs in Madagascar were predominantly caused by *E. coli* with prevalence rates varying from 57 to 70%, followed by other enterobacteria such as *Klebsiella pneumoniae* 9% to 11% [5, 6], *Enterobacter cloacae* 7% [5], and *Proteus mirabilis* 3% [6]. *Enterococcus faecalis*, one of the most frequent Gram positive bacterium in UTI next to *Staphylococcus saprophyticus* and *Streptococcus agalactiae*, was ranking with a prevalence of 4% [5]. On the other hand, the spread of bacteria expressing extended spectrum β-lactamases (ESBL) in both hospital and community settings becomes a worldwide problem. The CTX-M types (cefotaximases) confer resistance to 3rd generation cephalosporins (C3G) (i.e. cefotaxime, ceftriaxone, ceftazidime) and is the most widespread ESBL in the world [7–10]. During the period 2011–2013, Rasamiravaka et al*.* found 33% (77/234) of Gram-negative bacteria isolated from urine samples to be resistant to C3G in Antananarivo, Madagascar [11]. In 2016, the same prevalence rate of 33% (7/21) was found in urines, collected over a period of 4 months, of pregnant women with UTI attending the antenatal care clinic in the Hospital Center at Ambohimiandra, Antananarivo, Madagascar [12]. From January 2014 to October 2016, Rakotovao-Ravahatra et al*.*, reported a prevalence of 22.5% (N = 23) *E. coli* producing ESBLs among a total of 102 *E. coli* responsible for UTI diagnosed at the University Hospital Center Befelatanana, Antananarivo, Madagascar [13]. Among the C3G resistance genes, those from the CTX-M group 1 are the most common worldwide [9]. In 2007, a study carried out in hospitals and community care sites in Madagascar, revealed the presence of plasmids dominated by *bla-*CTX-M15 encoding for beta-lactam resistance (CTX-M group 1) in Enterobacteriaceae isolates [14]. Traditionally, the diagnosis of UTI is performed using dipstick tests, microscopic urinalysis, and culture-based pathogen detection. Urine dipsticks are easy to use, but false negative results are often obtained in the case of non-nitrite-producing pathogens, large amount of vitamin C, or diluted urine samples [15]. Microscopic urinalysis is a labor intensive and less sensitive method in particular for samples with bacteria concentrations below 10^5^ Colony Forming Units (CFU)/mL [16]. Urine culture, which is the gold standard for bacteria detection and identification, requires skilled laboratory technicians and has a long turnaround time from sample collection to result covering steps of isolation and identification of the microorganisms (usually between 18 and 48 h) followed by the determination of their susceptibility to antimicrobials (24 h). Among the new diagnostic methods, flow cytometry, matrix assisted laser desorption ionization–time of flight mass spectrometry (MALDI-TOF MS) and molecular diagnostics-based assays have recently been established to accelerate the identification of bacteria directly from urine samples. The flow cytometry-based urine analyzer, UF-5000 (Sysmex), has been reported to be a useful screening tool of Gram-negative bacteria in urine samples (sensitivity and specificity of 91.7% and 90.0%, respectively) but is limited to monobacterial infected urines containing ≥ 10^5^ CFU/mL, thus its utility depends highly on the patient population [17]. MALDI-TOF MS has been evaluated for its ability to identify pathogens directly in urine samples; however this requires a complex and time-consuming procedure like centrifugation and washing [18]. These two latter methods need a high investment cost and are therefore not appropriate in the context of low- and middle-income countries. Loop mediated isothermal amplification (LAMP), is a molecular method established in 2000 [19], and recognized by its characteristics of being simple, rapid and highly reliable. In addition, it is a very cost-effective diagnostic method. Consequently, it has increasingly been applied for the detection of disease/pathogens and genes of interest [20]. Recently, we developed a highly performant LAMP-based assay for the simple and rapid detection of the four most common CTX-M groups, namely CTX-M groups 1, 2, 8 and 9 [21]. In the present study, we report on the evaluation of a new LAMP *P. mirabilis* assay together with three previously described LAMP assays targeting *E. coli*, *K. pneumoniae*, *E. faecalis* in their effectiveness to the detect UTIs. In addition, we investigated the performance of the LAMP CTX-M group 1 assay detecting the most prevalent group of cefotaximases found in Madagascar.

## Methods

### Bacterial strains

The reference and clinical isolates are summarized in Table 1. Reference strains were provided by the National Reference Center (NRC) for antibiotic resistance, Paris, France. The clinical isolates of other species present in UTI were obtained from the Clinical Biology Center (CBC), Pasteur Institute of Madagascar.

**Table 1:** Bacteria used for the *P. mirabilis*-LAMP assay

Strains		Numbers of isolates tested	*P. mirabilis*-LAMP positive assay
Reference strains	*Proteus mirabilis* U2A 1878	1	1
	*Escherichia coli* U2A 1790	1	0
	*Klebsiella pneumoniae* U2A 759	1	0
	*Enterobacter cloacae *U2A 2242	1	0
	*Pseudomonas aeruginosa *U2A 1125	1	0
	*Acinetobacter baumannii* U2A 2479	1	0
	*Kluyvera georgiana*U2A 2251	1	0
	*Citrobacter freundii* 080320-170	1	0
	*Pantoea* spp*. *080326-0195	1	0
	*Serratia marcescens* 070223-087	1	0
	*Morganella morganii *061030-0203	1	0
	*Haemophillus influenzae*11-1717	1	0
	*Salmonella* spp. U2A 2145	1	0
	*Staphylococcus epidermidis *11-1819	1	0
	*Staphylococcus aureus *11-1669	1	0
	*Enterococcus faecalis *262 Bact. Med	1	0
	*Streptococcus agalactiae *127 Bact. Med	1	0
Clinical strains	*Proteus mirabilis*	38	38
	*Proteus vulgaris*	3	0
	*Escherichia coli*	4	0
	*Klebsiella pneumoniae*	4	0
	*Raoultella* sp.	1	0
	*Citrobacter freundii*	1	0
	*Citrobacter koseri*	1	0
	*Serratia marcescens*	1	0
	*Enterococus faecium*	1	0
	*Enterococcus faecalis*	2	0
	*Streptococcus agalactiae*	2	0

### DNA extracts

The strains were cultured using appropriate culture media and growth conditions. Two to three single colonies were suspended in 200 μL of distilled water (DW), placed into a boiling water bath for 10 min, subsequently transferred on ice for 5 min and then centrifuged at 20,000*g* for 5 min. The supernatant was used as template in the LAMP reactions.

### Urine samples

#### Spiked urine samples

Two to three colonies obtained by culture of the reference strains, including *E. coli, K. pneumoniae, P. mirabilis, E. faecalis* and *E. coli* producing a *bla-*CTX-M15 cefotaximase were suspended in bacteria free urine and, then incubated at 37 °C for 18 to 24H. Subsequently, urine suspensions of 0.5 MacFarland (McF) turbidity (= 1.5 10^8^ CFU/mL) were used and 100 µL of the suspensions were centrifuged at 20,000*g* for 5 min. The supernatant was removed and the pellets were re-suspended in 10 μL of DW. The sensitivities of the LAMP assays were determined by testing a range of 10-fold dilutions of the suspensions.

#### Urine samples from outpatients

A total of 166 human urine samples were obtained from the CBC between August 2016 and January 2017. A volume of 100 µL of each urine sample was centrifuged at 20,000*g* for 5 min and pellets were resuspended in 10 µL of DW. Five microliters (5 µL) of this suspension were used as template for LAMP assays. For each urine, the microbiological analysis was available.

### LAMP assays

#### LAMP primers

LAMP primers for *P. mirabilis* were designed by using the online Primer Explorer V4 software [22] and according to the general criteria described by Notomi et al*.* (2000)*.* The *P. mirabilis* target*, walR,* encodes an enzyme of the glycosyl transferase family involved in the biosynthesis of lipopolysaccharides [23]. The *walR* sequences were obtained from GenBank NCBI (www.ncbi.nlm.nih.gov/) and were tested in silico through BLAST searches. Alignment analysis of available analogue nucleotide sequences (Accession number ADK56074.1, HM146786.1, HM146785.1, HQ25931.1, HQ25930.1, KQ960958.1) were performed to design the following *P. mirabilis*-specific primers: F3 (forward outer primer), B3 (backward outer primer), FIP (forward inner primer), BIP (backward inner primer) and LF (loop forward primer). The specificity of the designed primers was confirmed by BLAST on the NCBI server. The primer sequences for the LAMP assays are shown in Table 2. The LAMP primers for *E. coli* [24], *K. pneumoniae* [25] and *E. faecalis* [26] were selected from the literature. LAMP primers for CTX-M group 1 detection were used as described previously [21].

**Table 2:** LAMP primer sets used in this study

Target	Primer name	Sequences (5′à 3′)	References
CTX-M groupe 1	F3	CACTGCGTCAGTTCACGC	Rivoarilala et al*.* [21]
	B3	CACGGCCATCACTTTACTGG	
	FIP	TTGCTGTACGTCCGCCGTTTGTTTTCAACCGTCACGCTGTTGT	
	BIP	CAGTCGGGAGGAAGACTGGGTTTTTGCGCTCATCAGCACGATA	
	LF	TACAGCGGCACACTTCCTA	
*P. mirabilis (walR)*	F3	AAAAAACGCGGWTCTGCA	This study
	B3	AAGACAGATAGAGCCAACG	
	FIP	CTGTCGAGCTATGGGTATTAATCACTTTTATTGCGTAATTGGTTAAAARTC	
	BIP	GTTAGTTGCGCTATCTTGTGCTTCTTTTGAACGTGATACATCGGTAGA	
	LF	CCGCCATAGTACGTACTCGCCA	
*E. coli*	F3	GCCATCTCCTGATGACG	Hill et al*.* [24]
	B3	ATTTACCGCAGCCAGACG	
	FIP	CTGGGGCGAGGTCGTGGTATTCCGACAAACACCACGAATT	
	BIP	CATTTTGCAGCTGTACGCTCGCAGCCCATCATGAATGTTGCT	
	LF	CTTTGTAACAACCTGTCATCGACA	
	LB	ATCAATCTCGATATCCATGAAGGTG	
*K. pneumoniae*	F3	GGATATCTGACCAGTCGG	Dong et al., [25]
	B3	GGGTTTTGCGTAATGATCTG	
	FIP	CGACGTACAGTGTTTCTGCAGTTTTAAAAAACAGGAAATCGTTGAGG	
	BIP	CGGCGGTGGTGTTTCTGAATTTTGCGAATAATGCCATTACTTTC	
	LB	GAAGACTGTTTCGTGCATGATGA	
*E. faecalis*	F3	GCCGGAAATCGATGAAGA	Kato et al*.* [26]
	B3	TCCAGCAACGTTGATTGT	
	FIP	CACTTTTTGTTGTTGGTTTTCGCTTTATTATCTGCTTGGGGTGC	
	BIP	ATCTGCAGACAAAGTAGTAATTGCTCCAAGCTTTTAAGCGTGTC	
	LF	AAATGCTGCGCCAGCTCG	
	LB	TCCAATGTGGAACTTAAACGTACC	

#### LAMP reaction

The LAMP reaction was performed as described previously [21]. Briefly, the reaction was carried out in a 25 μL mixture containing 0.2 μM of each outer primers F3 and B3, 0.8 μM of each inner primers FIP and BIP, and when available 0.4 μM of each loop primers LF or/and LB, 1X Thermopol Reaction Buffer (Biolabs), 0.8 M betaine, 7 mM MgSO_4_, 0.4 mM each deoxynucleotide triphosphate and 8U Bst DNA polymerase. Five microliter (5 μL) of DNA extract or sample was used as template in the reaction. Distilled water (DW) was used as negative control. The reaction was carried out in an Eppendorf® tube and incubated at 63 ± 2 °C in a thermo block for 60 min and was then heated at 80 ± 5 °C for 5 min to stop the reaction. The LAMP products were detected by direct visual inspection after addition of one microliter (1 µL) of Sybr Green I (SG I) 10 000X (http://www.sigmaaldrich.com) in the post reaction volume. A positive reaction was indicated by a change of color from orange to yellow, while a negative reaction had no change of color.

#### Analytical performance of the LAMP assays

The specificity of LAMP assays targeting *P. mirabilis, E. coli*, *K. pneumoniae* and *E. faecalis* were performed using DNA extracts obtained from 16 different bacterial species reference strains belonging to genera frequently isolated from UTI and 20 other anaerobic and facultative anaerobic strains isolated from UTI cases and particularly with 38 *P. mirabilis* clinical isolates for LAMP *P. mirabilis* (Table 1). Products were examined visually by staining with SG I and after electrophoresis on a 1.5% agarose gel—120 Volts—45 min. The assays were performed in triplicate to ensure its repeatability. The sensitivity of the five LAMP assays (*E. coli, K. pneumoniae, P. mirabilis, E. faecalis* and CTX-M group 1) was assessed in quintuplicate on a 10-fold serial dilution of spiked urine samples ranging from 10^1^ to 10^7^ CFU/mL. Products were examined visually by staining with SG I and analyzed by electrophoresis on a 1.5% agarose gel—120 Volts—45 min. The sensitivity was defined as the lowest concentration detected by the assay in four out of the five experiments.

#### Clinical performance of the five LAMP assays

One hundred microliter of the patient’s urine samples were centrifuged at 20,000*g* for 5 min. The pellets were suspended with 10 μL of DW. Five microliters of the suspension were used as template in each LAMP reaction. Products were examined visually by staining with SG I.

### Statistical methods

The diagnostic performance of the LAMP assays was reported as sensitivity, specificity, negative predictive value (NPV), positive predictive value (PPV) with 95% confidence interval (CI), considering culture as the gold standard. The agreement between diagnostic tests was calculated using Cohen’s kappa coefficient. Calculations were performed using Medcalc Easy software.

### Ethics

Ethical approval was not requested for this study because all clinical samples used were archived coded remnant samples provided by the CBC after all diagnostic assays in the context of the laboratory diagnosis of UTI were performed. The study was conducted anonymously.

## Results

### Analytical performance of the LAMP assays

**Fig. 1:**
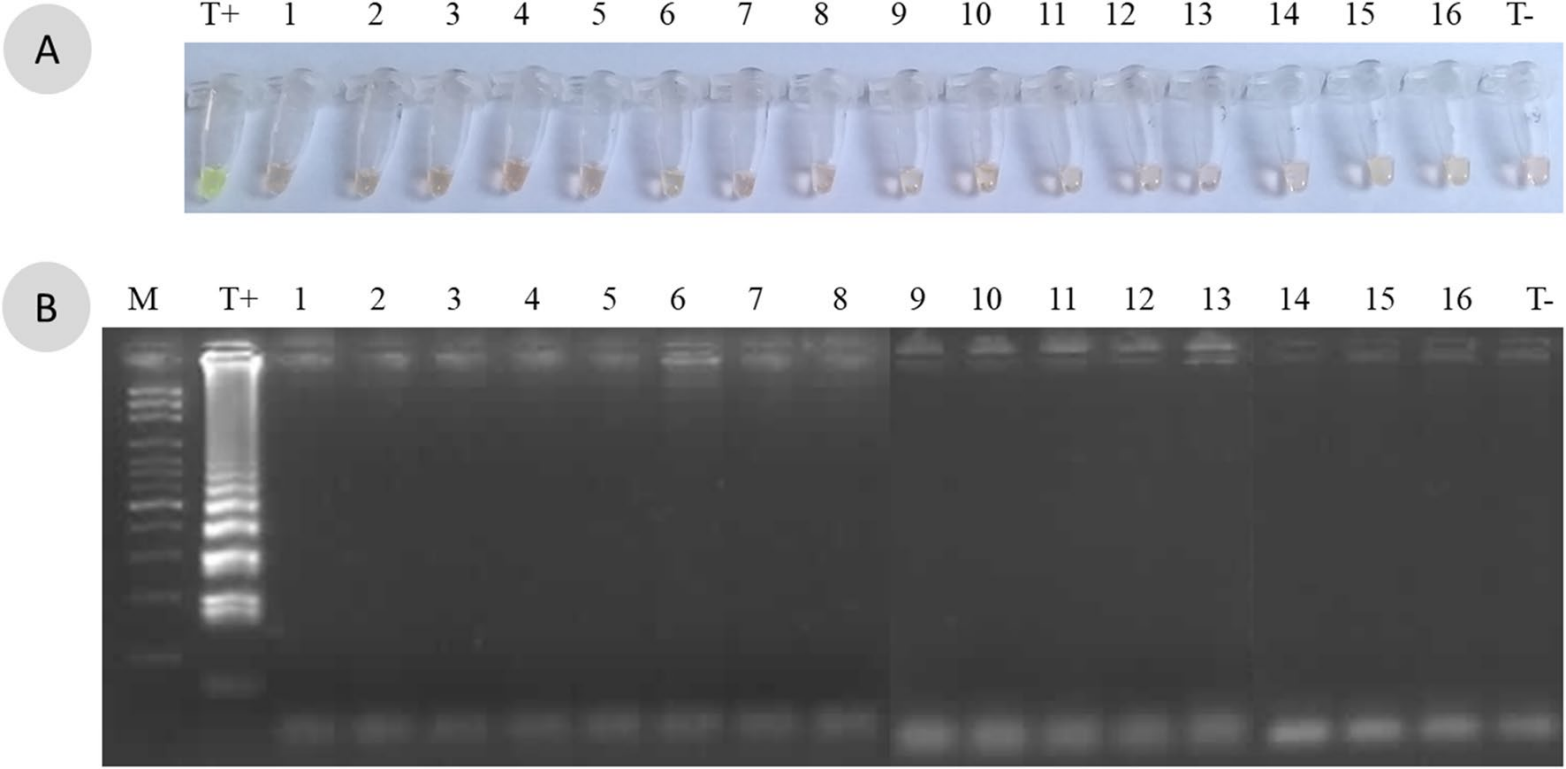
Specificity of the *P. mirabilis*-LAMP assay. **A** Staining with SG I. **B** Migration on agarose gel 1.5%. Tube and line: 1, *Acinetobacter baumannii* U2A 2479; 2, *E. coli* U2A 1790; 3, *Kluyvera georgiana* U2A 2251; 4, *Klebsiella pneumoniae* U2A 759; 5, *Pseudomonas aeruginosa* U2A 1125; 6, *E. cloacae* U2A 2242; 7, 080320-170 *Citrobacter freundii*; 8, *Morganellamorganii* 061030-0203; 9, *Pantoea* spp. 080326-0195; 10, *Haemophilus influenzae* 11-1717; 11, *Serratia marcescens* 070223-087; 12, *Salmonella* spp. U2A 2145; 13, *Enterococcus faecalis* 262 Bact. Med; 14, *Streptococcus agalactiae* 127 Bact. Med; 15, *Staphylococcus aureus* 11-1669; 16, *Staphylococcus epidermidis* 11-1819.—120 V—45 min. DNA ladder marker 100 bp; T + *, P. mirabilis* U2A 1878; T−, control (DW)

The BLAST analysis of the designed primers amplifying the *walR* gene from *P. mirabilis* did not show any mismatches. In addition, the *P. mirabilis* LAMP assay amplified only the DNA of the reference U2A 1878 and all 38 clinical strains of *P. mirabilis* (Table 1). No cross amplification was observed with the non-*P. mirabilis* reference (N = 16) strains (Fig. 1) (Additional files 1, 2, 3, 4; Fig. S1-S4. *The captions correspond to that of the Fig. 1*) nor with other clinical isolates causing UTIs (N = 20).

**Fig. 2:**
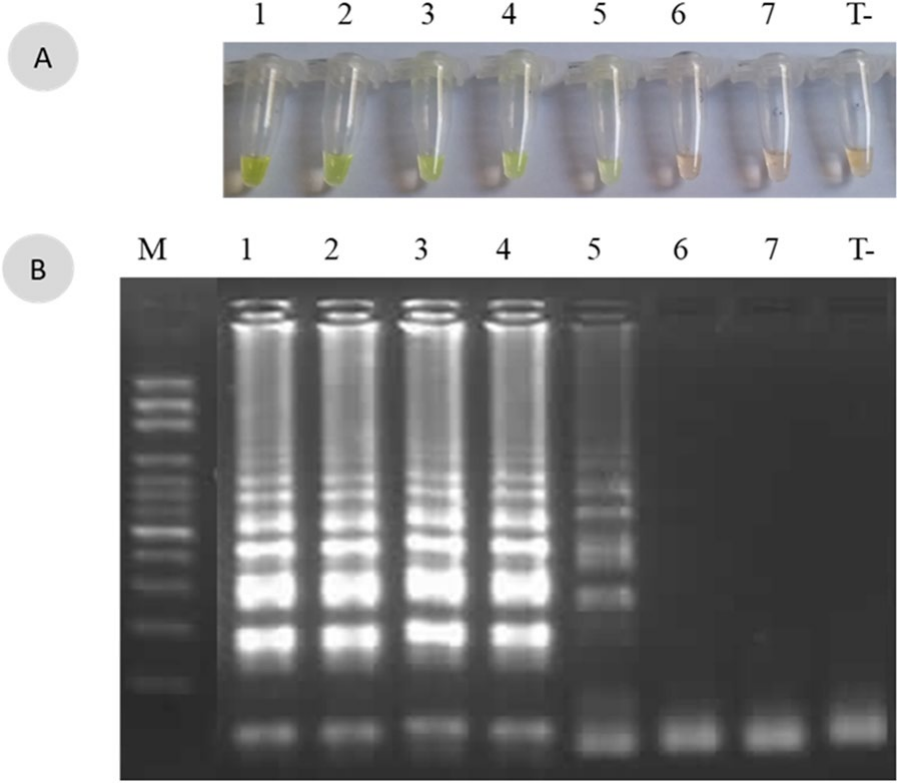
Sensitivity analysis of the loop mediated isothermal amplification (LAMP) for the detection of *P. mirabilis* on spiked urine samples. A range of 1.5 10-fold dilutions of pellet suspensions of *P. mirabilis* U2A 1878 were used. **A** Eye visualization of the LAMP reaction after coloration with the Sybr Green I dye. **B** Visualization after migration on an agarose gel of the LAMP products. M: marker, 1: 10^7^ CFU/mL, 2: 10^6^ CFU/mL, 3: 10^5^ CFU/mL, 4:10^4^ CFU/mL, 5: 10^3^ CFU/mL, 6: 10^2^ CFU/mL, 7: 10^1^ CFU/mL, T−, control (DW)

The specificity of the LAMP assays targeting *E. coli*, *K. pneumoniae* and *E. faecalis* was further confirmed by successful amplification of only the corresponding reference strains (data not shown). Positive SYBR Green I LAMP products or expected bands on agarose gels were only observed in the presence of the target DNA from reference strains. All triplicate results were concordant. The sensitivity for each assay was determined around 1.5 10^3^ CFU/mL (Fig. 2) (Additional files 5, 6, 7, 8; Fig. S5-S8. *The captions correspond to that of Fig. 2*).

### Clinical performance of LAMP assays

Out of the 166 urines collected and tested, five samples were excluded due to a polymicrobial population or a contaminated urine without a second sample analysis. The LAMP method was compared to bacterial culture, the gold standard, for 161 samples. The results are presented in Table 3. Using LAMP assays, *E. coli*, *K. pneumoniae*, *P. mirabilis*, and *E. faecalis* were detected in 15.5% (25/161), 8.7% (14/161), 1.9% (3/161), 2.0% (4/161) of the samples, respectively, while culture identified *E. coli* in 14.9% (24/161), *K. pneumoniae* in 3.7% (6/161), *P. mirabilis* in 1.9% (3/161) and *E. faecalis* in 1.2% (2/161) of the urine samples. Both techniques detected equally *P. mirabilis* (3/161) and the CTX-M group 1 genes (11/161) (Table 3). Note that all CTX-M group 1-LAMP positives were found resistant to C3G by culture and belonged to enterobacteria species such as *E. coli* (4/11), *K. pneumoniae* (3/11), *P. mirabilis* (1/11), *Enterobacter* spp. (1/11) and other Gram-negative bacteria such as *Acinetobacter ursinguii* (1/11) and *Citrobacter freundii* (1/11). The overall, sensitivity and specificity of LAMP assays ranged from 96 to 100% (CI: 15–100%) and from 95 to 100% (CI: 95–100%), respectively. The agreement between the LAMP assays and culture was the highest for *P. mirabilis* (K = 1) and the CTX-M group1 assays (K = 1), almost perfect for *E. coli* (K = 0,93), moderate for the *K. pneumoniae* (K = 0.58) and *E. faecalis* (K = 0.66) assays (Table 3).

**Table 3:** Comparison of culture and LAMP method results on 161 urine samples

LAMP	Culture		Sensitivity	Specificity	PPV	NPV	K
	Positive	Negative	% [95% CI]	% [95% CI]	% [95% CI]	% [95% CI]	
*E. coli*							
Positive	23 (TP)	2 (FP)	95.8	98.5	92	99.3	0.93
Negative	1 (FN)	135 (TN)	[78.9–99.9]	[94.8–99.8]	[74.4–97.9]	[95.2–99.9]	
*K. pneumoniae*							
Positive	6 (TP)	8 (FP)	100	94.8	42.9	100	0.58
Negative	0 (FN)	147 (VN)	[54.07–100]	[90.1–97.7]	[27.6–59.6]		
*P. mirabilis*							
Positive	3 (TP)	0 (FP)	100	100	100	100	1
Negative	0 (FN)	158 (TN)	[29.2–100]	[97.7–100]			
*E. faecalis*							
Positive	2 (TP)	2 (FP)	100	98.7	50	100	0.66
Negative	0 (FN)	157 (TN)	[15.8–100]	[95.5–99.8]	[20.1–79.8]		
*CTX-M groupe 1*							
Positive	11 (TP)	0 (FP)	100	100	100	100	1
Negative	0 (FN)	150 (TN)	[71.5–100]	[97.6–100]			

CI, Confidence Interval; TP, true positive, FP, false positive; TN, true negative; FN, false negative; PPV, positive predictive value; NPV, negative predictive value; K, Cohen’s Kappa

## Discussion

We developed and evaluated several LAMP assays for the simple and rapid detection of clinically important uropathogens (i.e. *E. coli*, *K. pneumoniae*, *E. faecalis*, *P. mirabilis*) and genes encoding the most widespread cefotaximases (CTX-M group 1, including CTX-15 the most widespread cefotaximase detected in Madagascar).

The overall sensitivity of the LAMP assays was around 1.5 10^3^ CFU/mL which corresponds to the threshold of bacteriuria (10^3^ CFU/mL for bacteria such as *E. coli*, enterobacteria other than *E. coli* and *Enterococcus*) associated with a leucocyturia > 10^4^/mL in symptomatic patients [1]. This sensitivity was 10 to 100 time higher than that of the study of Etchebarne BE et al*.* (2017) which was 1.10 10^5^ CFU/mL for LAMP targeting *E. coli*, 50 10^3^ CFU/mL for *K. pneumoniae*, 150 10^3^ CFU/mL for *E. faecalis* and 20 10^3^ CFU/mL for *P. mirabilis* [27]. However, Etchebarne BE et al*.*, performed a DNA extraction by heating.

In our study, we have demonstrated that a LAMP assay applied to urine samples without an extraction step is possible. The five LAMP assays we tested herein were all found specific. The side-by-side performance of LAMP and culture was based on the test of 161 urine samples. The LAMP assays were able to detect all *K. pneumoniae*, *E. faecalis*, *P. mirabilis* and CTX-M group 1 resistance genes, but missed one *E. coli* in one infected urine*.* The LAMP assays detected additionally two *E. coli*, eight *K. pneumoniae* and two *E. faecalis* infected urines, which may be explained by the higher analytical sensitivity of the LAMP assays compared to culture results.. From practical point of view, this LAMP method is simple and rapid. It did not require a complex DNA extraction step. LAMP assays provided a significantly shorter turnaround time compared to culture (1h30 vs 3 days).

Comparing to other studies, the specificity values of our LAMP assays to detect *E. coli* (99%), *K. pneumoniae* (95%) and *E. faecalis* (99%) were close to those found by Rödel et al*.* (2017), 97% (n = 157) for *E. coli*, 99% (n = 157) for *K. pneumoniae* and 98% (n = 71) for *E. faecalis*. It is worthwhile to mention that the latter assays were performed in blood culture and the amplification were carried out using the eazyplex® commercial test and Genie II machine [28]. Additionally, Etchebarne et al*.* (2017) found similar results for overall specificities (97%), using the culture as a reference method [27]. When compared to culture of urine, we obtained eight false positive results with the *K. pneumoniae*-LAMP assay on urines which we could not investigate in this study. Similarly, Dong et al*.* (2012) observed seven false positive results among 110 sputa in wich *K. pneumoniae* was detected using PCR as reference method [29]. However, they and others confirmed the expected target sequences after sequencing the LAMP products [29–31] suggesting that the false positives were most probably true positives and thus adding evidence to the superior sensitivity of the LAMP assay [32, 33]. However, LAMP assays are prone to primer dimer, hairpin structure formation and non-specific amplification. These can be avoided by adding dimethylsulfoxide (DMSO) to the reaction. De-Guo Wang et al. (2015) and Kinyatta Nanc et al. (2021) demonstrated that adding 7.5% of DMSO in the reaction mix improved the LAMP specificity [34, 35].The potential benefit of the LAMP assays developed in this study is their ability to detect not only bacteria responsible for UTI but also the most common resistance to C3G antibiotics, the CTX-M group 1. The early diagnosis of this gene could lead to improving the antibiotic therapy and antimicrobial stewardship (Additional file 1, Additional file 2, Additional file 3, Additional file 4).

We did not include DNA extraction steps; simplifying considerably the procedure. LAMP assays have shown a significant tolerance to amplification inhibiting substances derived from a number of biological samples including urine [36–38]. Thereby, some LAMP assays with high sensitivity and simple procedure for DNA extraction have been developed for molecular detection of bacterial [39] and parasitic [40] infections in urine samples. Additionally, a simple DNA extraction method by heating has been successfully applied with other types of clinical samples, such as blood in the LAMP assay for *Plasmodium* [41] and cutaneous swabs for the detection of *Leishmania* species [42]. In the study of Gandasegui et al*.* (2015), among the three DNA extraction methods tested, the higher performance of the LAMP assay for *S. haematobium* detection in urine samples was obtained after heating the pellet obtained after centrifugation of the samples and using it as template for the LAMP reaction. The heating step was necessary to free, besides *S. haematobium* DNA, DNA from the parasite eggs [43]. Based on the previous studies, we performed a centrifugation and a heat treatment as a simple pre-treatment of samples. In addition, the LAMP reaction was performed in a water bath and did not require a thermal cycler. The amplification products were detected visually by observing a color change without the need of any other equipment. Finally, the turnaround time of analysis including preparation of the sample, the LAMP assay and the interpretation of the products was around 90 min. All these characteristics advocate the use of this method as a simple, inexpensive and performant assay adapted to the settings encountered in low- and middle-income countries. However, some disadvantages of the LAMP assay need to be acknowledged. LAMP is not useful for cloning. The primer design is subject to constrains, such as the reduced freedom to choose the target site and the requirement of free, open-source [26] or commercial software packages. The multiplexing approaches for multiple pathogens’ detection are very complex and poorly still developed [44]. Since LAMP is a highly sensitive method, detection of the end products by adding dye in a post-amplification step implying the opening of the tubes could increase the risk of aerosol contamination if it is not done in an adequate manner. Previous studies demonstrated that this issue of contamination can be solved by the use of other dyes added to the reagent mix in the pre-amplification step, such as the Malachite Green (MG), Crystal Violet, Calcein, Hydroxynaphtol Blue, Phenol Red, Fluorescent Dye [45, 46]. Among these, MG was recently reported as the best indicator of visualization of LAMP products [46]. In addition, this dye is cheap, the color is stable, easy to use, and its storage conditions are appropriate for use in low and middle income countries. Moreover, two novel methods named Recombinase Assisted Loop-mediated Amplification (RALA) and ladder-shape melting temperature isothermal amplification (LMTIA) could be interesting approaches. RALA combines the advantages of the strand displacement activity of recombinase and the high efficiency of loop-mediated amplification. The nucleoprotein complex formed by the primers and the recombinase opens the dsDNA at the target site and initiates the amplification. In addition, the introduction of a special probe for sequence-specific real-time detection could increase further the specificity and reproducibility of the RALA amplification [47]. LMTIA is based on the amplification of targets characterized by ladder-type melting temperature curves. The method uses nested primers and a thermostable DNA polymerase. According to the authors the method is highly specific and sensitive thanks to the specific primer sequences and primers’ melting temperature, and the particular requirement of the target melting temperature curve [48]. In order to diagnose, treat and manage UTIs, besides the presence of bacteria in a concentration > 10^3^ CFU/mL, the leucocyturia, the aspects of the urine and symptoms of the patient has to be taken into account. Besides, other pathogens such as *Enterobacter* spp., *Citrobacter* spp and *Staphylococcus* spp. could be responsible for UTI and those are not yet covered in our current assays. In our perspective, the UTI-LAMP assays were developed as alternative tools for health facilities in low-income countries where culture cannot be implemented. However, if culture is done in routine it should not be disregarded and the LAMP assays could be implemented as a complement to culture with the aim to accelerate the detection of resistant pathogens. Additionally, a quantitative approach could be used, performing real-time turbidity measurements of the LAMP reaction and extrapolating the time to result with the CFU/mL of the target pathogen [29, 49] (Additional file 5, Additional file 6, Additional file 7, Additional file 8).

## Conclusion

The LAMP method may be used as an alternative or complementary tool to culture for early detection of common uropathogens and resistance genes from the CTX-M group 1 adapted to resource-limited settings.

## Supplementary Information

**Additional file 1. Figure S1:** The original figure of Figure 1A. Specificity of the *P. mirabilis*-LAMP assay. **A** Staining with SG I. **B** Migration on agarose gel 1.5%. Tube and line: 1, *Acinetobacter baumannii* U2A 2479; 2, *E. coli* U2A 1790; 3, *Kluyvera georgiana* U2A 2251; 4, *Klebsiella pneumoniae* U2A 759; 5, *Pseudomonas aeruginosa* U2A 1125; 6, *E. cloacae* U2A 2242; 7, 080320-170 *Citrobacter freundii*; 8, *Morganellamorganii* 061030-0203; 9, *Pantoea* spp. 080326-0195; 10, *Haemophilus influenzae* 11-1717; 11, *Serratia marcescens* 070223-087; 12, *Salmonella* spp. U2A 2145; 13, *Enterococcus faecalis* 262 Bact. Med; 14, *Streptococcus agalactiae* 127 Bact. Med; 15, *Staphylococcus aureus* 11-1669; 16, *Staphylococcus epidermidis* 11-1819.—120 V—45 min. DNA ladder marker 100 bp; T + *, P. mirabilis* U2A 1878; T−, control (DW)

**Additional file 2. Figure S1.2:** The original, unprocessed version of the Figure 1A.

**Additional file 3. Figure S1.3:** The original, unprocessed version of the Figure 1A.

**Additional file 4. Figure S1.4:** The original version of the Figure 1A adding brightness 15%.

**Additional file 5. Figure S2.1:** The figure original of Figure 2A. Sensitivity analysis of the loop mediated isothermal amplification (LAMP) for the detection of *P. mirabilis* on spiked urine samples. A range of 1.5 10-fold dilutions of pellet suspensions of *P. mirabilis* U2A 1878 were used. **A** Eye visualization of the LAMP reaction after coloration with the Sybr Green I dye. **B** Visualization after migration on an agarose gel of the LAMP products. M: marker, 1: 10^7^ CFU/mL, 2: 10^6^ CFU/mL, 3: 10^5^ CFU/mL, 4:10^4^ CFU/mL, 5: 10^3^ CFU/mL, 6: 10^2^ CFU/mL, 7: 10^1^ CFU/mL, T−, control (DW)

**Additional file 6. Figure S2.2:** The original unprocessed version of the Figure 2B.

**Additional file 7. Figure S2.2:** The original figure of Figure 2B. The marker on the right is the one that is more visualized and used in the Figure 2.

**Additional file 8. Figure S2.3:** The same figure of S2.2 adding in the left the best marker.

## Data Availability

The datasets used and/or analysed during the current study are available from the corresponding author on reasonable request. The sequences for the analysis were obtained from GenBank NCBI www.ncbi.nlm.nih.gov/ (Accession number ADK56074.1, HM146786.1, HM146785.1, HQ25931.1, HQ25930.1, KQ960958.1).

## Abbreviations

UTIs: Urinary tract infections
LAMP: Loop mediated isothermal amplification
CTX-M: Cefotaximases
C3G: 3Rd Generation cephalosporins
CFU: Colony forming units
MALDI-TOF MS: Matrix assisted laser desorption ionization–time of flight mass spectrometry
CBC: Clinical Biology Center Clinical
DW: Distilled water
SG I: Sybr Green I
NPV: Negative predictive value
PPV: Positive predictive value
CI: Confidence interval
