# Supplementary figures and images for: LAMP assays for the simple and rapid detection of clinically important urinary pathogens including the detection of resistance to 3rd generation cephalosporins
Source: BMC Infect Dis. 2021 Oct 6;21:1037. doi: 10.1186/s12879-021-06720-5 (PMC8495977; doi:10.1186/s12879-021-06720-5)

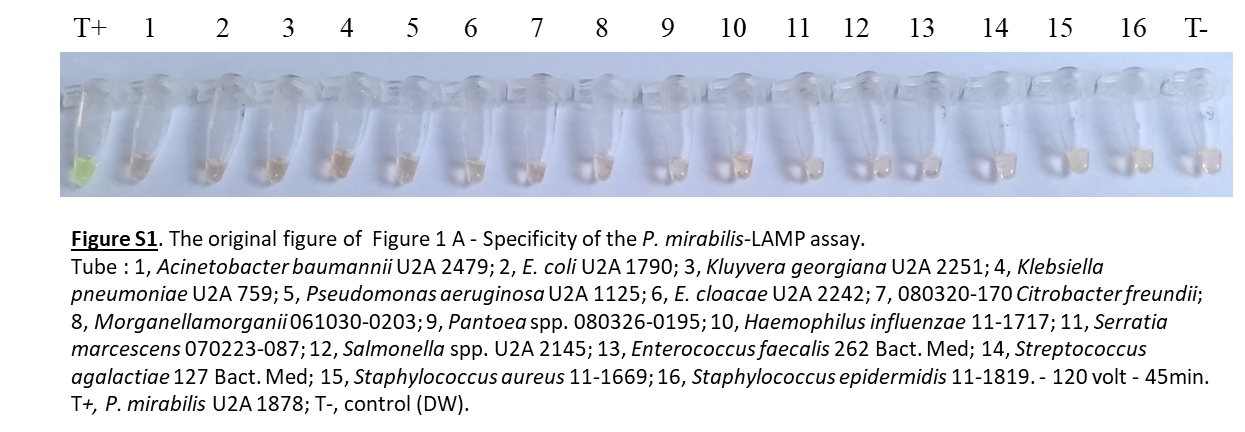

Supplement: Supplementary file 1 — Additional file 1. Figure S1: The original figure of Figure 1A. Specificity of the P. mirabilis-LAMP assay. A Staining with SG I. B Migration on agarose gel 1.5%. Tube and line: 1, Acinetobacter baumannii U2A 2479; 2, E. coli U2A 1790; 3, Kluyvera georgiana U2A 2251; 4, Klebsiella pneumoniae U2A 759; 5, Pseudomonas aeruginosa U2A 1125; 6, E. cloacae U2A 2242; 7, 080320-170 Citrobacter freundii; 8, Morganellamorganii 061030-0203; 9, Pantoea spp. 080326-0195; 10, Haemophilus influenzae 11-1717; 11, Serratia marcescens 070223-087; 12, Salmonella spp. U2A 2145; 13, Enterococcus faecalis 262 Bact. Med; 14, Streptococcus agalactiae 127 Bact. Med; 15, Staphylococcus aureus 11-1669; 16, Staphylococcus epidermidis 11-1819.—120 V—45 min. DNA ladder marker 100 bp; T + , P. mirabilis U2A 1878; T−, control (DW) [file 12879_2021_6720_MOESM1_ESM.png]

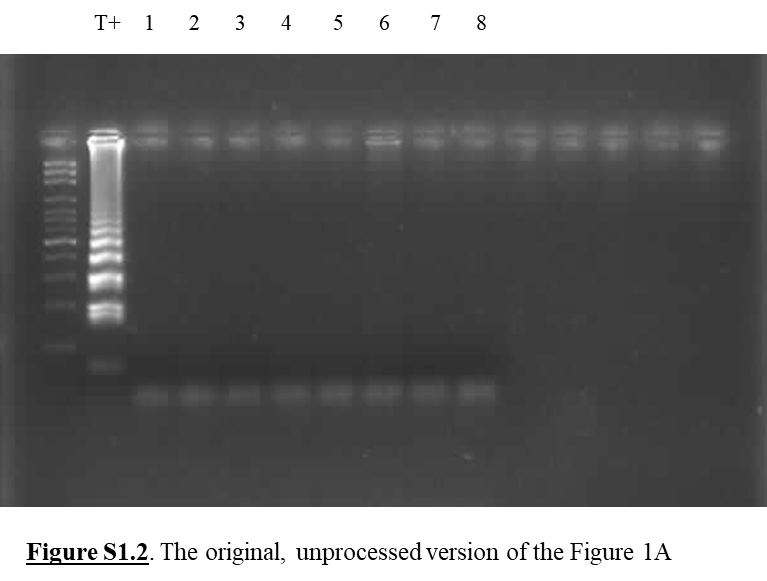

Supplement: Supplementary file 2 — Additional file 2. Figure S1.2: The original, unprocessed version of the Figure 1A. [file 12879_2021_6720_MOESM2_ESM.png]

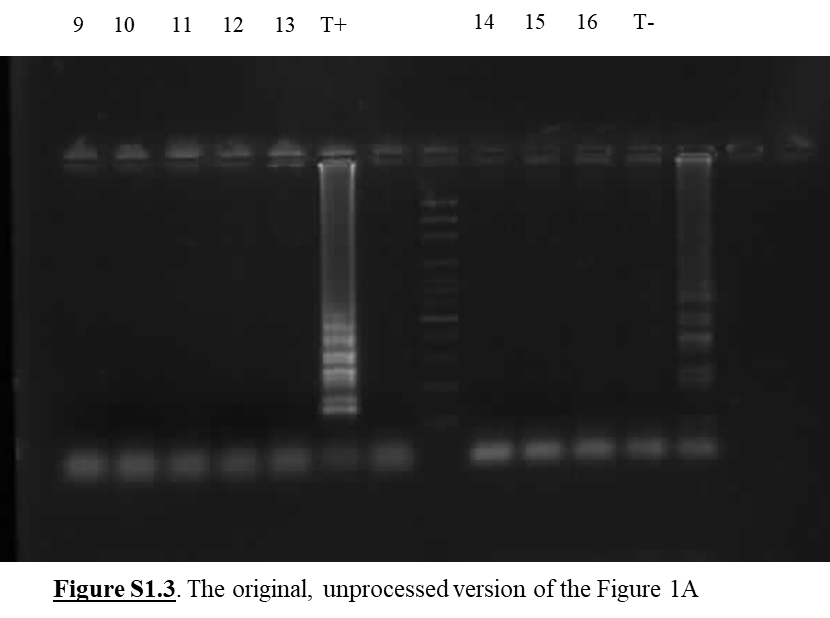

Supplement: Supplementary file 3 — Additional file 3. Figure S1.3: The original, unprocessed version of the Figure 1A. [file 12879_2021_6720_MOESM3_ESM.png]

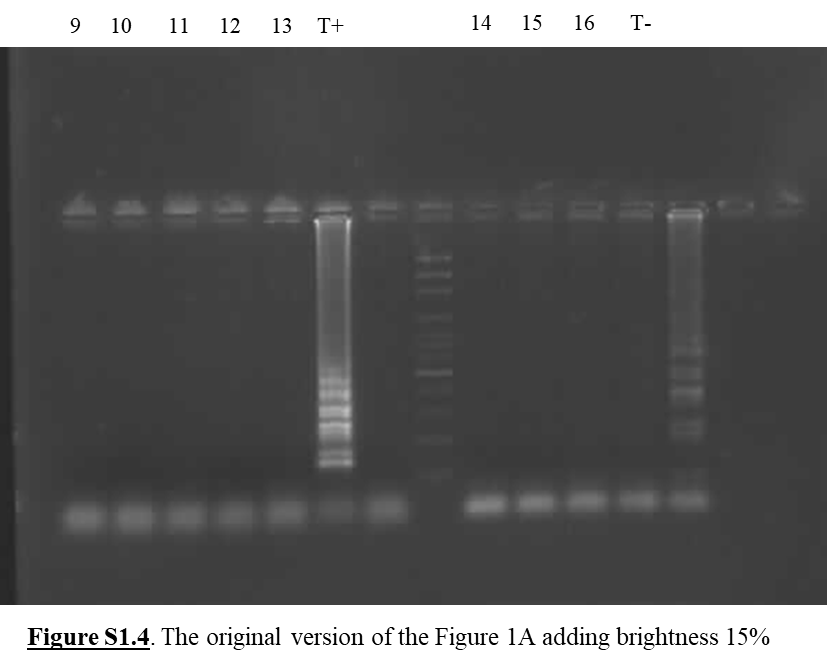

Supplement: Supplementary file 4 — Additional file 4. Figure S1.4: The original version of the Figure 1A adding brightness 15%. [file 12879_2021_6720_MOESM4_ESM.png]

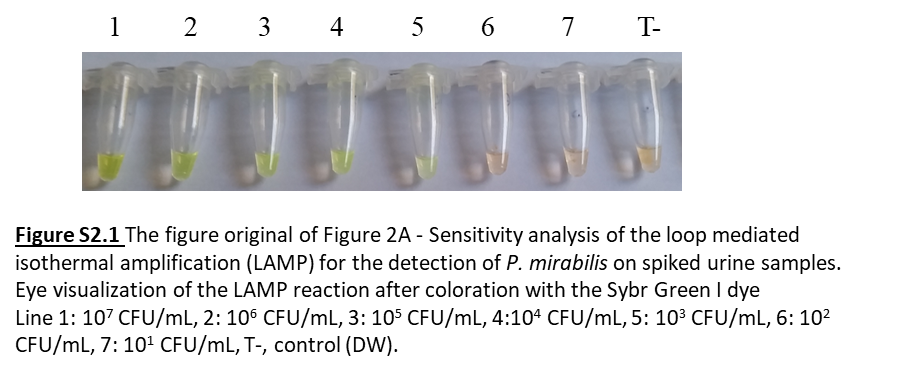

Supplement: Supplementary file 5 — Additional file 5. Figure S2.1: The figure original of Figure 2A. Sensitivity analysis of the loop mediated isothermal amplification (LAMP) for the detection of P. mirabilis on spiked urine samples. A range of 1.5 10-fold dilutions of pellet suspensions of P. mirabilis U2A 1878 were used. A Eye visualization of the LAMP reaction after coloration with the Sybr Green I dye. B Visualization after migration on an agarose gel of the LAMP products. M: marker, 1: 107 CFU/mL, 2: 106 CFU/mL, 3: 105 CFU/mL, 4:104 CFU/mL, 5: 103 CFU/mL, 6: 102 CFU/mL, 7: 101 CFU/mL, T−, control (DW) [file 12879_2021_6720_MOESM5_ESM.png]

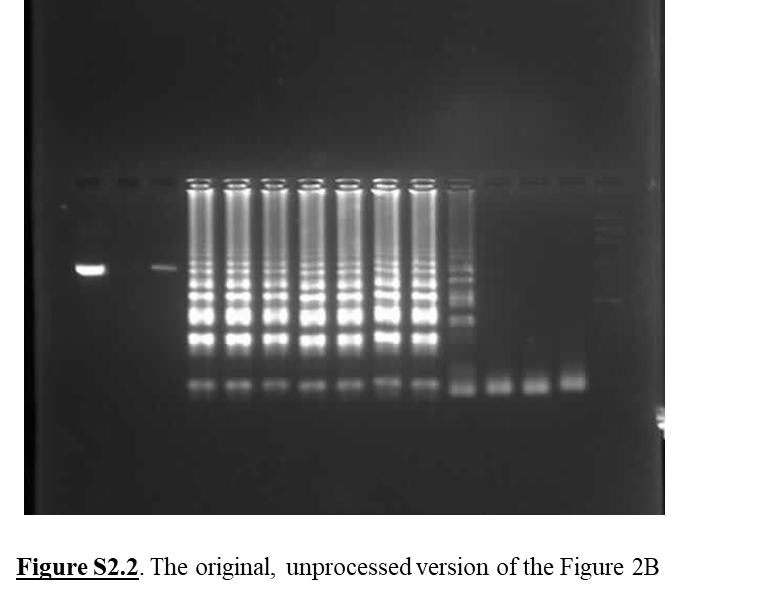

Supplement: Supplementary file 6 — Additional file 6. Figure S2.2: The original unprocessed version of the Figure 2B. [file 12879_2021_6720_MOESM6_ESM.png]

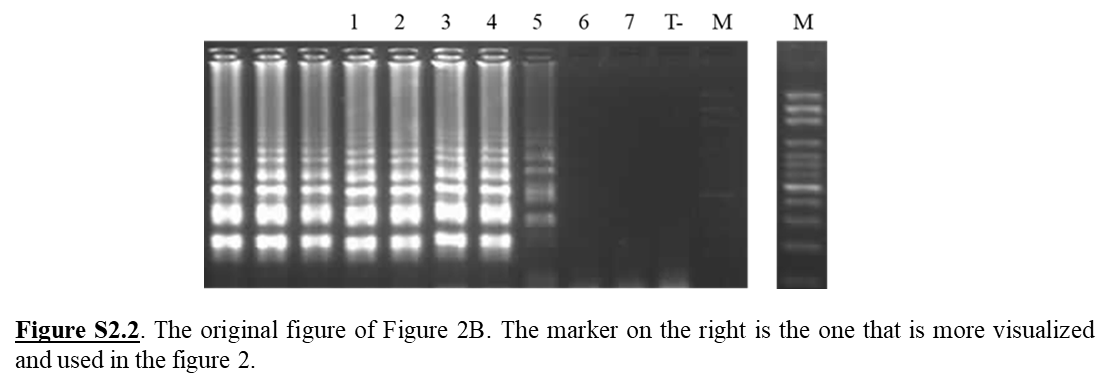

Supplement: Supplementary file 7 — Additional file 7. Figure S2.2: The original figure of Figure 2B. The marker on the right is the one that is more visualized and used in the Figure 2. [file 12879_2021_6720_MOESM7_ESM.png]

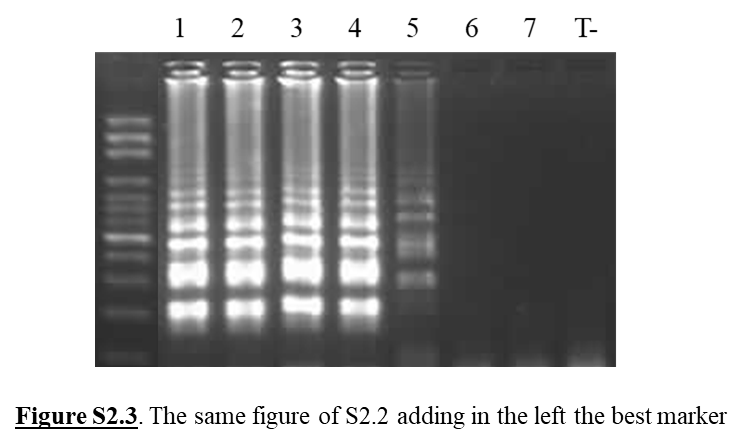

Supplement: Supplementary file 8 — Additional file 8. Figure S2.3: The same figure of S2.2 adding in the left the best marker. [file 12879_2021_6720_MOESM8_ESM.png]
